# Risk of diabetes mellitus among users of immune checkpoint inhibitors: A population‐based cohort study

**DOI:** 10.1002/cam4.5616

**Published:** 2023-01-16

**Authors:** Jeffrey Shi Kai Chan, Sharen Lee, Dicken Kong, Ishan Lakhani, Kenrick Ng, Edward Christopher Dee, Pias Tang, Yan Hiu Athena Lee, Danish Iltaf Satti, Wing Tak Wong, Tong Liu, Gary Tse

**Affiliations:** ^1^ Cardio‐Oncology Research Unit, Cardiovascular Analytics Group Hong Kong China; ^2^ Department of Medical Oncology University College London Hospitals NHS Foundation Trust London UK; ^3^ Department of Radiation Oncology Memorial Sloan Kettering Cancer Center New York New York USA; ^4^ School of Life Sciences The Chinese University of Hong Kong Hong Kong China; ^5^ Tianjin Key Laboratory of Ionic‐Molecular Function of Cardiovascular Disease, Department of Cardiology Tianjin Institute of Cardiology, Second Hospital of Tianjin Medical University Tianjin China; ^6^ Kent and Medway Medical School University of Kent and Canterbury Christ Church University Canterbury, Kent UK; ^7^ School of Nursing and Health Studies Hong Kong Metropolitan University Hong Kong China

**Keywords:** competing risk, CTLA‐4, diabetes, immune checkpoint inhibitors, PD‐1, PD‐L1

## Abstract

**Background:**

Immune checkpoint inhibitors (ICIs) are increasingly established cancer therapeutics, but they are associated with new‐onset diabetes mellitus (DM). Such risks have not been adequately quantified, and between‐class and ‐sex differences remain unexplored.

**Methods:**

This was a prospective cohort study of cancer patients receiving any ICI in Hong Kong between 2013 and 2021. Patients with known DM were excluded. Due to few patients using other ICIs, only programmed cell death 1 inhibitors (PD‐1i) and programmed death ligand 1 inhibitors (PD‐L1i) were compared, alongside between‐sex comparison. When comparing PD‐1i against PD‐L1i, patients with the use of other ICIs or both PD‐1i and PD‐L1 were further excluded. Inverse probability treatment weighting (IPTW) was used to minimize between‐group covariate imbalances.

**Results:**

Altogether, 3375 patients were analyzed (65.2% males, median age 62.2 [interquartile range 53.8–69.5] years old). Over a median follow‐up of 1.0 [0.4–2.4] years, new‐onset DM occurred in 457 patients (13.5%), with a 3‐year risk of 14.5% [95% confidence interval 13.3%, 15.8%]. IPTW achieve acceptable covariate balance between sexes, and between PD‐1i (*N* = 622) and PD‐L1i (*N* = 2426) users. Males had significantly higher risk of new‐onset DM (hazard ratio 1.35 [1.09, 1.67], *p* = 0.006), while PD‐1i and PD‐L1i users did not have significantly different risks (hazard ratio vs PD‐L1i 0.81 [0.59, 1.11], *p* = 0.182). These were consistent in those with at least 1 year of follow‐up, and on competing risk regression.

**Conclusion:**

Users of ICI may have a substantial risk of new‐onset DM, which may be higher in males but did not differ between PD‐1i and PD‐L1i.

## Graphical abstract

This population‐based study from Hong Kong quantified the burden of new‐onset diabetes mellitus among patients with cancer receiving ICIs. Such risk may be higher in males, but may not differ significantly between PD‐1 inhibitor and PD‐L1 inhibitor users.

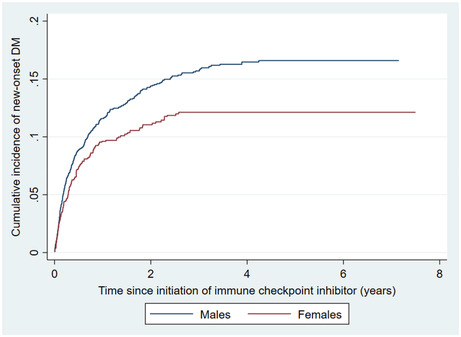

## 1 INTRODUCTION

Over the past decade, immunotherapy has revolutionized the field of oncology, providing novel, efficacious alternatives to conventional management strategies in the treatment of numerous advanced‐staged malignancies.^1^ Immune checkpoint inhibitors (ICIs) are monoclonal antibodies that function by relieving the tumor‐mediated inhibition on T‐cell activity, thereby amplifying the antitumor capacity of such immune cells to suppress metastatic progression and improve long‐term patient outcomes. In particular, ICIs target the cytotoxic T lymphocyte antigen 4 (CTLA‐4)‐CD28 axis or the programmed cell death 1 (PD‐1) – programmed death ligand 1 (PD‐L1) axis. With PD‐1 protein being highly expressed on the surface of activated T cells, overexpression of PD‐L1 allows some malignant cells to evade immunosurveillance mechanisms through PD‐1/PD‐L1 interaction, which may be prevented by PD‐1 and PD‐L1 inhibitors (PD‐1i and PD‐L1i).^2^

However, the role of the PD‐1/PD‐L1 pathway in the maintenance and regulation of immune tolerance implies that PD‐1i/PD‐L1i may lead to a loss of self‐tolerance,^3^ resulting in various immune‐related adverse events. Meanwhile, CTLA‐4 inhibitors (CTLA‐4i) may cause immune‐related adverse events via a number of complex pathways, including but not limited to increased T cell cytokine receptor expressions, and T cell hyper‐responsiveness due to reduced attenuation of major histocompatibility complex receptor signaling on T cells.^4^, ^5^ Immune‐related adverse events may also relate to abnormal activation of B cells and thus generation of autoantibodies,^5^ such as antibodies against islet cell antigens and glutamic acid decardoxylase‐65 which may cause diabetes mellitus (DM).^6^, ^7^ Overall, CTLA4i have more widespread and rapid effects due to their activation of T nodes from lymph nodes and their “enhancement” of immune responses,^5^, ^8^, ^9^ contrasting the predominant modulation of peripheral T cells by PD1i and PDL1i and their “normalization” of immune responses.^5^, ^8^, ^9^ Such differences also meant that the types of toxicities differ between CTLA4i and PD1i / PDL1i, with the former predominantly associated with inflammation of colon, skin, and pituitary, and the latter predominantly associated with inflammation of thyroid, muscles, joints, lungs, and skin.^8^

Owing to the autoimmune nature of these events, they may affect a wide range of organ systems, resulting in cutaneous, gastrointestinal, pulmonary, hepatic, neurological, cardiac, and endocrine manifestations.^10^, ^11^, ^12^, ^13^, ^14^ As above mentioned, these include DM,^6^, ^7^, ^15^, ^16^ which, given its well‐known detrimental effects on multiple organ systems, may add substantially to the morbidity of patients using ICIs. Nonetheless, as a relatively new endocrinopathy, the risk of new‐onset DM in patients receiving ICIs has not been adequately quantified, and it remains unclear whether there are differences in the risk of new‐onset DM between different types of ICIs. Additionally, while studies have suggested that the efficacy of ICIs may differ between sexes, it remains unclear whether the risk of new‐onset DM among users of ICIs exhibits such differences.^17^, ^18^ Therefore, this study aimed to quantify the risk of developing DM in cancer patients treated with ICIs, and to compare the risk of new‐onset DM between sexes and different types of ICIs.

## 2 METHODS

This study was approved by The Joint Chinese University of Hong Kong—New Territories East Cluster Clinical Research Ethics Committee. It was in line with the Strengthening the Reporting of Observational Studies in Epidemiology guideline and the Declaration of Helsinki. The requirement for individual patient consent was waived as only retrospective, deidentified data were used. All underlying data are available on reasonable request to the corresponding authors.

### 2.1 Data source

Data were extracted from the Clinical Data Analysis and Reporting System (CDARS), a prospective, population‐based electronic medical database capturing the basic demographics, diagnoses, laboratory investigations, and medical procedures of all patients attending public hospitals and clinics in Hong Kong, which cover the whole territory of Hong Kong and serve approximately 90% of the population.^19^ CDARS records diagnoses using the International Classification of Diseases, Ninth revision (ICD‐9) codes, as ICD‐10 codes have not been used in CDARS to date. Mortality data were acquired from the linked Hong Kong Death Registry, a governmental mortality registry of Hong Kong citizens. Both databases have been used in previous studies, and have been demonstrated to have good coding accuracy and data completeness.^20^, ^21^, ^22^, ^23^, ^24^

### 2.2 Patient population

All patients with cancer receiving any ICI in Hong Kong between January 1, 2013 and December 31, 2021 were identified. ICI included PD‐1i (pembrolizumab or nivolumab), PD‐L1i (atezolizumab, avelumab, or durvalumab), and CTLA‐4i (ipilimumab); other ICI were not available in Hong Kong when this study was conducted. Patients with prior diagnosis of DM were excluded from the descriptive analysis of the burden of new‐onset DM.

As very few patients ever used CTLA‐4i, only PD‐1i and PD‐L1i were compared in this study. In this comparative analysis, patients who ever used both PD‐1i and PD‐L1i (including non‐concurrent prescriptions), and those who used CTLA‐4i were further excluded. No exclusion criteria were applied for the between‐sex comparative analysis.

### 2.3 Follow‐up and outcome

All patients were followed up until December 31, 2021. The outcome of interest was new‐onset DM. Throughout this study, DM was defined by ICD‐9 codes (Table S1), usage of any anti‐diabetic medication, any HbA1c measurement ≥6.5%, or any two consecutive fasting glucose measurements ≥7.0 mmol/L.^25^

### 2.4 Data collected

The following data were collected for all patients: demographics (age and sex), type of cancer, comorbid conditions (hypertension (defined by both ICD‐9 codes and use of antihypertensive(s)), ischemic heart disease, myocardial infarction, heart failure, atrial fibrillation, dyslipidemia (defined by both ICD‐9 codes and use of anti‐lipid medication(s)), chronic obstructive pulmonary disease, and stroke), and use of non‐ICI medications (angiotensin‐converting enzyme inhibitors or angiotensin receptor blockers, beta‐blockers, statins, dihydropyridine calcium channel blockers, chemotherapy, and steroids). There were no missing data.

### 2.5 Statistical analyses

Continuous variables were expressed as median with interquartile ranges. The incidence rate of new‐onset DM was estimated and described. The cumulative incidence of new‐onset DM was modeled and visualized using the Aalen–Johansen method, with consideration of mortality as a competing event. Correspondingly, the 1‐year, 2‐year, and 3‐year risks of new‐onset DM were estimated and described.

In the separate comparisons between males and females and between PD‐1i and PD‐L1i, inverse probability treatment weighting (IPTW) was used to minimize imbalances in baseline characteristics between groups. The propensity scores were calculated using the *twang* package, with which a generalized boosted model with a maximum of 10,000 regression trees and an iteration stopping point minimizing the absolute standardized mean difference of the mean effect size were used, with all possible three‐way interactions allowed. All recorded baseline covariates listed above were included in the IPTW, and were summarized in Table 1 (male vs. female) and Table S2 (PD‐1i vs.PD‐L1i). Standardized mean difference (SMD) was used to quantify inter‐group balance of covariates, with SMD <0.2 considered to represent acceptable balance. The cause‐specific cumulative incidence of new‐onset DM was modeled and visualized using the Aalen–Johansen method, with consideration of mortality as competing event.^26^, ^27^ The Aalen–Johansen method was used instead of the Kaplan–Meier method, as the latter is known to over‐estimate cumulative incidences in the presence of competing risks.^28^ Univariable Cox regression with IPTW was used to compare treatment groups, with hazard ratio (HR) with 95% confidence interval as summary statistics.

**TABLE 1:** Characteristics of included patients, and balance in characteristics between male and female patients before and after inverse probability treatment weighting (IPTW).

	All patients	Females	Males	Pre‐IPTW SMD	Post‐IPTW SMD
Number of patients, *N*	3375	1173	2202	NA	NA
*Demographics*					
Age, years [interquartile range]	62.2 [53.8–69.5]	59.7 [50.9–67.5]	63.3 [55.6–70.3]	0.264	0.028
Male, *N* (%)	2202 (65.2)	0 (0)	2202 (100)	NA	NA
*Type of immune checkpoint inhibitor used*					
PD‐1i, *N* (%)	2749 (81.5)	958 (81.7)	1791 (81.3)	0.009	0.010
PD‐L1i, *N* (%)	691 (20.5)	237 (20.2)	454 (20.6)	0.010	0.018
CTLA‐4i, *N* (%)	269 (8.0)	90 (7.7)	179 (8.1)	0.017	0.022
*Type of cancer*					
Head and neck cancers, *N* (%)	126 (3.7)	38 (3.2)	88 (4.0)	0.040	0.024
Lung cancer, *N* (%)	1595 (47.3)	510 (43.5)	1085 (49.3)	0.116	0.033
Melanoma, *N* (%)	90 (2.7)	46 (3.9)	44 (2.0)	0.119	0.035
Renal cell carcinoma, *N* (%)	124 (3.7)	31 (2.6)	93 (4.2)	0.084	0.025
*Comorbid conditions*					
Hypertension, *N* (%)	1292 (38.3)	405 (34.5)	887 (40.3)	0.118	0.019
Ischemic heart disease, *N* (%)	105 (3.1)	15 (1.3)	90 (4.1)	0.162	0.059
Myocardial infarction, *N* (%)	25 (0.7)	5 (0.4)	20 (0.9)	0.056	0.009
Heart failure, *N* (%)	25 (0.7)	6 (0.5)	19 (0.9)	0.041	0.019
Atrial fibrillation, *N* (%)	61 (1.8)	21 (1.8)	40 (1.8)	0.002	0.011
Dyslipidemia, *N* (%)	647 (19.2)	175 (14.9)	472 (21.4)	0.166	0.038
COPD, *N* (%)	86 (2.6)	3 (0.3)	83 (3.8)	0.223	0.117
Stroke, *N* (%)	43 (1.3)	14 (1.2)	29 (1.3)	0.011	0.007
*Medications used*					
ACEI/ARB, *N* (%)	509 (15.1)	136 (11.6)	373 (16.9)	0.149	0.046
Dihydropyridine CCB, *N* (%)	1018 (30.2)	307 (26.2)	711 (32.3)	0.133	0.029
Beta‐blocker, *N* (%)	594 (17.6)	194 (16.5)	400 (18.2)	0.043	0.002
Statin, *N* (%)	590 (17.5)	153 (13.0)	437 (19.9)	0.179	0.051
Chemotherapy, *N* (%)	2038 (60.4)	782 (66.7)	1256 (57.0)	0.197	0.040
Steroid, *N* (%)	1354 (40.1)	534 (45.5)	820 (37.2)	0.169	0.019

Abbreviations: ACEI, angiotensin‐converting enzyme inhibitor; ARB, angiotensin receptor blocker; CCB, calcium channel blocker; COPD, chronic obstructive pulmonary disease; CTLA‐4i, cytotoxic T‐lymphocyte‐associated protein 4 inhibitor; NA, not applicable; PD‐1i, programmed cell death protein‐1 inhibitors; PD‐L1i, programmed death ligand‐1 inhibitors; SMD, standardized mean difference.

Two a priori sensitivity analyses were done for each comparison. First, as DM occurring soon after ICI initiation may be due to premorbid conditions instead, a sensitivity analysis was performed limiting to those with at least 1 year of follow‐up. Second, a sensitivity analysis was performed using Fine and Gray competing risk regression with the sub‐distribution model to account for mortality; the sub‐hazard ratio with 95% confidence interval was used as summary statistics.

All *p*‐values were two‐sided, and *p* < 0.05 was considered statistically significant. All statistical analyses were performed on Stata v16.1 (StataCorp LLC, Texas, USA).

## 3 RESULTS

**FIGURE 1:**
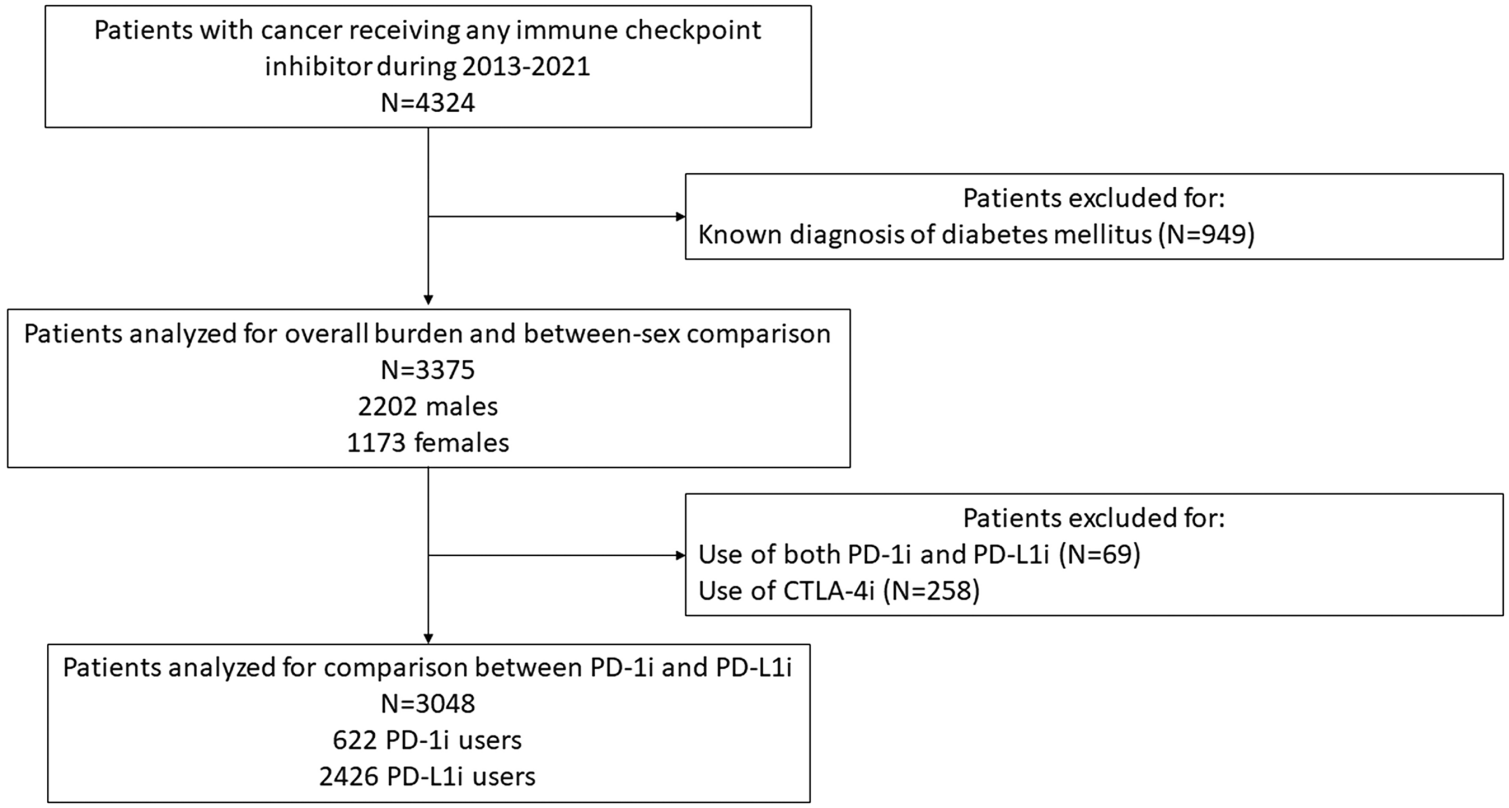
Study flowchart. CTLA‐4i, cytotoxic T‐lymphocyte‐associated protein 4 inhibitors. PD‐1i, programmed cell death protein‐1 inhibitors. PD‐L1i, programmed death ligand‐1 inhibitors.

In total, 4324 ICI users were identified. After excluding 949 patients with known DM (Figure 1), 3375 were analyzed (2202 (65.2%) males, median age 62.2 [IQR 53.8–69.5] years old), of whom 2426 (71.9%) only ever used PD‐1i, 622 (18.4%) only ever used PD‐L1i, four (0.1%) only ever used CTLA‐4i (0.1%), and 323 (9.6%) used more than one ICI. Almost half of the patients had lung cancer (1595 patients, 45.3%). Baseline characteristics of the included patients are summarized in Table 1.

### 3.1 Overall burden of new‐onset DM in ICI users

**FIGURE 2:**
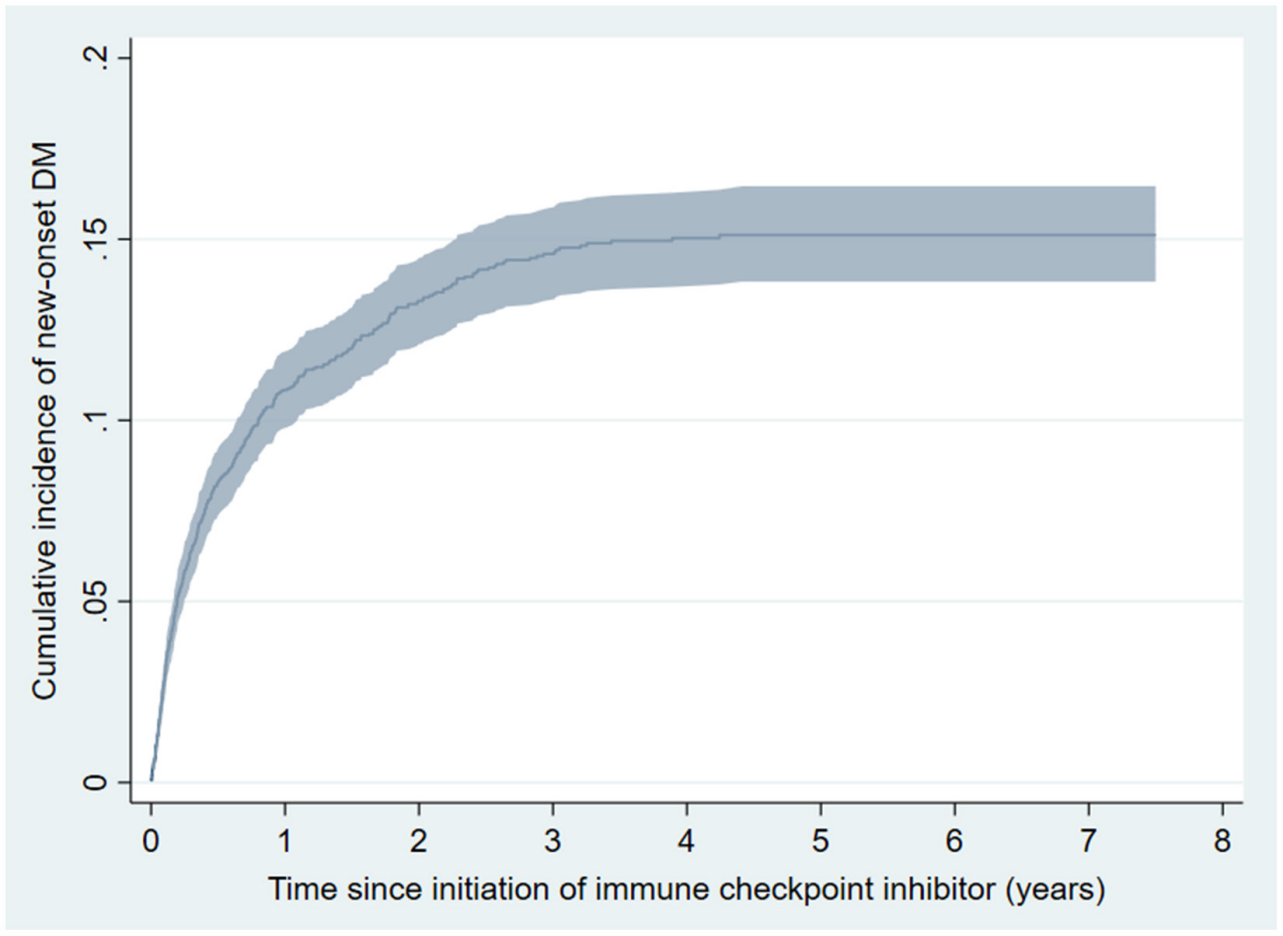
Cumulative incidence curve of new‐onset diabetes mellitus (DM) throughout the study period. The shaded area indicates the 95% confidence interval.

Over a median follow‐up of 1.0 [0.4–2.4] years, new‐onset DM occurred in 457 patients (13.5%), while 1563 (46.3%) died without having new‐onset DM. The incidence rate of new‐onset DM was 8.6 [7.8, 9.4] cases per 100 person‐years. Among those who developed new‐onset DM, the median time to new‐onset DM was 125 [43–311] days. The cause‐specific cumulative incidence of new‐onset DM is visualized in Figure 2, with the 1‐year risk being 10.8% [95% confidence interval 9.8%, 11.9%], the 2‐year risk being 13.3% [12.1%, 14.5%], and the 3‐year risk being 14.5% [13.3%, 15.8%].

### 3.2 Comparing the risk of new‐onset DM between sexes

**FIGURE 3:**
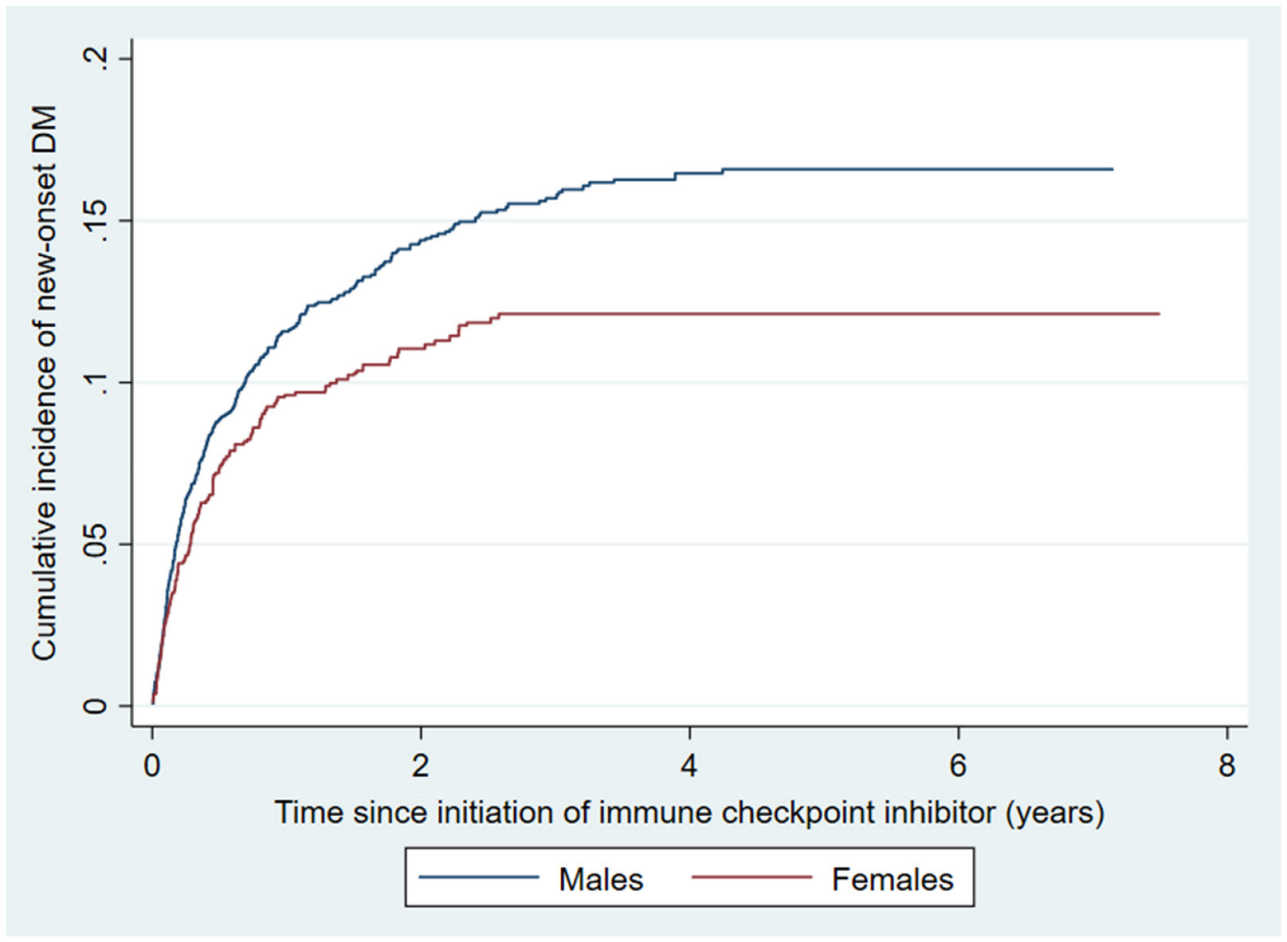
Cumulative incidence curve of new‐onset diabetes mellitus (DM) among male and female patients throughout the study period.

During follow‐up, new‐onset DM occurred in 326 males (14.8%) and 131 females (11.2%). IPTW achieved acceptable balance of covariates between groups (SMD <0.2; Table 1). Males had significantly higher risk of new‐onset DM (HR 1.35 [1.09, 1.67], *p* = 0.006; Figure 3). Such observation was consistent on limiting the analysis to patients with at least 1 year of follow‐up (*N* = 1705; HR 1.50 [1.10, 2.03], *p* = 0.010), and on competing risk regression (sub‐hazard ratio 1.34 [1.08, 1.66], *p* = 0.008).

### 3.3 Comparing the risk of new‐onset DM for users of PD‐1i and PD‐L1i

As only four patients only ever used CTLA‐4i, only PD‐1i and PD‐L1i were compared for the risk of new‐onset DM. After further excluding 69 users of both PD‐1i and PD‐L1i and 258 users of CTLA‐4i (Figure 1), 3048 patients were analyzed (622 PD‐1i users and 2426 PD‐L1i users; median age 62.6 [54.5–69.8] years old; 1984 (65.1%) males). IPTW of covariates achieved acceptable balance between treatment groups (SMD <0.2; Table S2).

**FIGURE 4:**
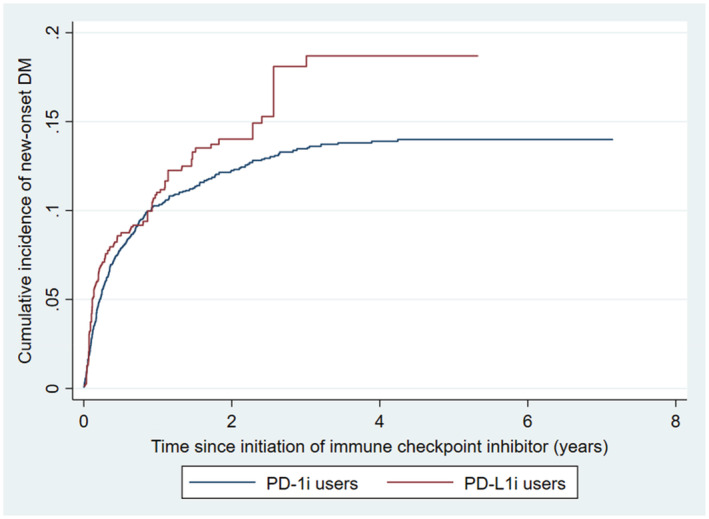
Cumulative incidence curve of new‐onset diabetes mellitus (DM) among users of PD‐1i (programmed cell death protein‐1 inhibitors) and PD‐L1i (programmed death ligand‐1 inhibitors) throughout the study period.

Over a median follow‐up of 1.0 [0.4–2.3] years, new‐onset DM occurred in 306 PD‐1i users (12.6%) and 85 PD‐L1 users; 1399 died without developing new‐onset DM (45.9%). Users of PD‐1i and PD‐L1i did not differ significantly in the risk of new‐onset DM (HR 0.81 [0.59, 1.11], *p* = 0.182 referencing against PD‐L1i; Figure 4), which was consistently observed on limiting the analysis to those with at least 1 year of follow‐up (*N* = 1505; HR 0.64 [0.39, 1.04], *p* = 0.073), and on competing risk regression (sub‐hazard ratio 0.89 [0.66, 1.21], *p* = 0.476).

## 4 DISCUSSION

In this population‐based study, we quantified the risk of new‐onset DM among patients with cancer receiving ICIs, with consideration given to mortality as competing risk. We showed that such risks may be higher in males but may not differ significantly between PD‐1i and PD‐L1i users.

Previous studies focusing on ICI‐related DM have either been case reports or series,^29^, ^30^ relied on data from pharmacovigilance databases,^31^, ^32^, ^33^ or adopted a case‐only approach where only patients with ICI‐related DM were studied.^31^, ^34^ As such designs are often heavily affected by selection bias and that underreporting of events is common in pharmacovigilance databases, these findings may be seen as hypothesis‐generating and may not be representative. Some have performed post hoc analysis or meta‐analysis of randomized controlled trials.^33^ While these would be ideal for comparing ICI against other therapies in terms of the risk of DM, most trials only include highly selected populations, implying that such findings are unlikely to be representative or generalizable to real‐world practice. In contrast, we analyzed data from a population‐based database that essentially included all patients that have ever received any ICI in Hong Kong, ensuring that our findings closely reflect real‐world practice. We observed that new‐onset DM has an overall incidence rate of 8.6 cases per 100 person‐years. Putting these numbers in context, it was estimated that in Hong Kong, type 2 DM had an incidence rate of approximately 0.5–1 and 1–2 cases per 100 person‐years among those aged 40–59 years old and those aged at least 60 years old, respectively, while the corresponding incidence rates for type 1 DM were essentially negligible for these age groups.^35^ The substantially higher incidence rate of new‐onset DM observed in this study echoed previous findings that ICI use is associated with elevated risks of DM.^36^

Furthermore, we observed that males had an estimated 35% higher risk of new‐onset DM, which was confirmed on two sensitivity analyses. However, as we did not specifically analyze immune‐related DM, our findings may have been driven at least in part by the known, higher risk of DM in males,^37^, ^38^ which is attributable to endogenous estrogen being protective of DM, as well as probable between‐sex differences in energy partitioning, energy balance, and body composition.^38^ While this was supported by a 2021 meta‐analysis showing no significant difference between sex in immune‐related adverse events among ICI users, several more recent studies have suggested otherwise with somewhat conflicting results.^39^, ^40^ Overall, potential between‐sex differences in the risk of new‐onset DM remains contentious and incompletely understood, and further investigations are warranted. On the other hand, we found no significant difference in the risk of new‐onset DM between PD‐1i and PD‐L1i, which was consistent with a previous meta‐analysis by Lu and colleagues.^36^ Unfortunately, very few patients had received CTLA‐4i in Hong Kong at the time of this study being conducted, preventing relevant comparisons. A meta‐analysis has suggested that CTLA‐4i may carry higher risk of immune‐related adverse events than PD‐1i and PD‐L1i.^41^ Further studies comparing CTLA‐4i with other classes of ICIs for the risk of new‐onset DM are warranted.

Our findings allow clinicians to have a better understanding of the risk of new‐onset DM in patients with cancer receiving ICIs. It is essential the risks related to ICI use are clearly communicated to patients prior to ICI initiation,^42^ and our population‐based statistics should aid clinicians in such discussions and facilitate share decision‐making. Nonetheless, in addition to the between‐sex and between‐class differences that we have alluded to above, many gaps remain in the understanding of DM in ICI users, including its risk factors, tools for early detection and risk stratification, prognosis, and management. In particular, although ICI‐related DM has mainly been described as an acute condition,^43^, ^44^ emerging evidence suggested that long‐term or delayed‐onset adverse events may occur.^45^, ^46^ Also, without any non‐ICI‐exposed group, this study was not designed to investigate any incremental long‐term risk of new‐onset DM associated with ICI use, and more detailed quantification in this regard remains needed. Overall, further studies of the long‐term associations between ICIs and DM are warranted, and larger studies in different populations are required to validate our findings.

### 4.1 Strengths and limitations

Having utilized data from a population‐based database, our results are representative of ICI users in Hong Kong, and are likely generalizable to other Asian regions. Additionally, we used appropriate statistical tools to properly address the impact of mortality as a competing risk which may otherwise skew estimates of cumulative incidences.^28^ Nonetheless, this study was not devoid of limitations. First, due to the nature of the database, the data could not be individually adjudicated. This meant that it was not possible to differentiate immune‐related DM from DM due to other causes (e.g., old age), which limited the interpretation of our findings. Nevertheless, our findings still provided a broad picture of the burden of new‐onset DM in patients with cancer receiving ICIs which, regardless of the exact etiology and subtype of new‐onset DM, remains a valuable information for clinicians and patients alike. The lack of adjudication was also unlikely to significantly affect the validity of our data, as data input was performed by treating clinicians independent of the authors, and both the CDARS and the Hong Kong Death Registry have been demonstrated to have good data accuracy and completeness.^20^ Second, due to the nature of the database used, cancer staging and histological subtypes were not available. Third, inherent to the observational nature of this study, there may be residual or unobserved confounders. Nonetheless, we have attempted to minimize these limitations by considering as many potential confounders as possible in IPTW.

## 5 CONCLUSIONS

In this population‐based study, we quantified the burden of new‐onset DM among patients with cancer receiving ICIs. The risk of new‐onset DM may be higher in males, but may not differ significantly between PD‐1i and PD‐L1i users. Further studies are required to confirm our findings.

### LAY SUMMARY

Patients receiving immune checkpoint inhibitors have significant risks of new‐onset diabetes, which may be higher in males but may not differ between specific classes of these medications.

## AUTHOR CONTRIBUTIONS

**Jeffrey Shi Kai Chan:** Conceptualization (equal); data curation (lead); formal analysis (lead); methodology (lead); visualization (lead); writing – original draft (lead); writing – review and editing (lead). **Sharen Lee:** Conceptualization (equal); investigation (equal); writing – review and editing (supporting). **Dicken Kong:** Investigation (equal); writing – review and editing (supporting). **Ishan Lakhani:** Investigation (equal); writing – review and editing (supporting). **Kenrick Ng:** Supervision (equal); writing – review and editing (supporting). **Edward Christopher Dee:** Supervision (equal); writing – review and editing (supporting). **Pias Tang:** Investigation (equal); writing – review and editing (supporting). **Yan Hiu Athena Lee:** Investigation (equal); writing – review and editing (supporting). **Danish Iltaf Satti:** Investigation (supporting); writing – review and editing (supporting). **Wing Tak Wong:** Supervision (equal); writing – review and editing (supporting). **Tong Liu:** Funding acquisition (lead); supervision (equal); writing – review and editing (supporting).

## FUNDING INFORMATION

This work was funded by the Tianjin Key Medical Discipline (Specialty) Construction Project (Project number: TJYXZDXK‐029A). The funder played no role in any part of this study.

## CONFLICTS OF INTEREST

ECD is funded in part through the Cancer Center Support Grant from the National Cancer Institute (P30 CA008748). None of the other authors had any relationship with industry or conflict of interest.

## PATIENT CONSENT STATEMENT

Requirement for individual patient consent was waived as only retrospective data were used.

## ETHICAL APPROVAL

This study was approved by The Joint Chinese University of Hong Kong – New Territories East Cluster Clinical Research Ethics Committee.

## Supporting information


Table S1

## Data Availability

All underlying data is available on reasonable request to the corresponding authors.
