# Supplementary material for: Risk of diabetes mellitus among users of immune checkpoint inhibitors: A population‐based cohort study
Source: Cancer Med. 2023 Jan 16;12(7):8144–53. doi: 10.1002/cam4.5616 (PMC10134274; doi:10.1002/cam4.5616)
Supplement: Supplementary file 1 — Table S1 [file CAM4-12-8144-s001.docx]

**Supplementary Material**

**Supplementary Table 1.** International Classification of Diseases, Ninth revision (ICD-9) codes used for identifying diagnoses. All listed codes include the corresponding sub-codes.

| **Condition** | **ICD-9 diagnostic codes** |
| --- | --- |
| Diabetes mellitus | 250 |
| Lung cancer | 162.3-162.9 |
| Head and neck cancer | 140-149.9 |
| Melanoma | 172-172.9 |
| Renal cell carcinoma | 189 |
| Myocardial infarction | 410-411.0, 412 |
| Heart failure | 428, 402.01, 402.11, 402.91 |
| Stroke | 430, 431-432, 433.01, 433.11, 433.21, 433.31, 433.81, 433.91, 434.01, 434.11, 434.91, 435 |
| Hypertension | 401-405, 437.2 |
| Ischaemic heart disease | 410-414 |
| Atrial fibrillation | 427.31 |
| Dyslipidaemia | 272.0-272.4 |

**Supplementary Table 2.** Balance in covariates between users of programmed cell death protein-1 inhibitors (PD-1i) and programmed death ligand-1 inhibitors (PD-L1i) before and after inverse probability treatment weighting (IPTW).

|  | PD-1i users | PD-L1i users | Pre-IPTW SMD | Post-IPTW SMD |
| --- | --- | --- | --- | --- |
| Number of patients, N | 2426 | 622 | NA | NA |
| *Demographics* | | | | |
| Age, years [interquartile range] | 62.3 [53.6-69.9] | 63.4 [56.8-69.5] | 0.125 | 0.046 |
| Male, N (%) | 1575 (64.9) | 409 (65.8) | 0.017 | 0.036 |
| *Type of cancer* | | | | |
| Head and neck cancers, N (%) | 114 (4.7) | 7 (1.1) | 0.183 | 0.106 |
| Lung cancer, N (%) | 1017 (41.9) | 519 (83.4) | 0.830 | 0.125 |
| Melanoma, N (%) | 76 (3.1) | 0 (0) | 0.201 | 0.164 |
| Renal cell carcinoma, N (%) | 100 (4.1) | 2 (0.3) | 0.211 | 0.145 |
| *Comorbid conditions* | | | | |
| Hypertension, N (%) | 950 (39.2) | 212 (34.1) | 0.104 | 0.082 |
| Ischaemic heart disease, N (%) | 74 (3.1) | 19 (3.1) | 0 | 0.029 |
| Myocardial infarction, N (%) | 19 (0.8) | 2 (0.3) | 0.056 | 0.064 |
| Heart failure, N (%) | 21 (0.9) | 2 (0.3) | 0.063 | 0.067 |
| Atrial fibrillation, N (%) | 48 (2.0) | 11 (1.8) | 0.015 | 0.022 |
| Dyslipidaemia, N (%) | 477 (19.7) | 125 (20.1) | 0.011 | 0.009 |
| COPD, N (%) | 59 (2.4) | 26 (4.2) | 0.106 | 0.009 |
| Stroke, N (%) | 32 (1.3) | 7 (1.1) | 0.017 | 0.003 |
| *Medications used* | | | | |
| ACEI/ARB, N (%) | 371 (15.3) | 84 (13.5) | 0.050 | 0.009 |
| Dihydropyridine CCB, N (%) | 743 (30.6) | 171 (27.5) | 0.068 | 0.053 |
| Beta-blocker, N (%) | 449 (18.5) | 98 (15.8) | 0.072 | 0.073 |
| Statin, N (%) | 434 (17.9) | 113 (18.2) | 0.007 | 0.015 |
| Chemotherapy, N (%) | 1346 (55.5) | 530 (85.2) | 0.611 | 0.123 |
| Steroid, N (%) | 933 (38.5) | 300 (48.2) | 0.199 | 0.016 |

ACEI, angiotensin-converting enzyme inhibitor. ARB, angiotensin receptor blocker. CCB, calcium channel blocker. COPD, chronic obstructive pulmonary disease. NA, not applicable. PD-1i, programmed cell death protein-1 inhibitors. PD-L1i, programmed death ligand-1 inhibitors. SMD, standardized mean difference.
